# Decision support system for *Lespedeza cuneata* production and quality evaluation: a WebGIS dashboard approach to precision agriculture

**DOI:** 10.3389/fpls.2025.1520163

**Published:** 2025-07-17

**Authors:** Sudhanshu S. Panda, Aftab Siddique, Thomas H. Terrill, Ajit K. Mahapatra, Eric Morgan, Andres A. Pech-Cervantes, Jan A. van Wyk

**Affiliations:** ^1^ Institute for Environmental Spatial Analysis, University of North Georgia, Oakwood, GA, United States; ^2^ Department of Agricultural Sciences, Fort Valley State University, Fort Valley, GA, United States; ^3^ Institute for Global Food Security, Queen’s University, Belfast, United Kingdom; ^4^ International Goat Research Center, College of Agriculture, Food and Natural Resources, Prairie View A&M University, Texas, TX, United States; ^5^ Department of Veterinary Tropical Diseases, Faculty of Veterinary Science, University of Pretoria, Onderstepoort, South Africa

**Keywords:** site-specific fodder management, sericea lespedeza, geographic information systems, remote sensing, precision agriculture

## Abstract

Small-scale farmers in the southeastern United States face increasing challenges in sustaining forage production due to erratic rainfall, poor soils, and limited access to precision agricultural tools. These constraints demand site-specific solutions that integrate climate resilience with sustainable land use. This study introduces a pioneering Site-Specific Fodder Management Decision Support System (SSFM-DSS) designed to optimize the cultivation of *Lespedeza cuneata* (sericea lespedeza), a drought-tolerant, nitrogen-fixing legume well-suited for marginal lands. By integrating high-resolution geospatial technologies—Geographic Information Systems (GIS), Global Navigation Satellite Systems (GNSS), and remote sensing—with empirical field data and predictive modeling, we have developed an automated suitability framework for SL cultivation across Alabama, Georgia, and South Carolina. The model incorporates multi-criteria environmental parameters, including soil characteristics, topography, and climate variability, to generate spatially explicit recommendations. To translate these insights into actionable strategies, we also developed a farmer-focused WebGIS Dashboard that delivers real-time, location-based guidance for SL production. Our findings underscore the significant potential of SSFM-DSS to enhance fodder availability, improve system resilience under climate stress, and promote sustainable livestock production. This integrative approach offers a promising pathway for climate-smart agriculture, supporting broader food security objectives in vulnerable agroecosystems.

## 1 Introduction

*Lespedeza cuneata* (Dum.-Cours.) G. Don., sericea lespedeza (SL), is a warm-season perennial legume adapted to the soil types and climatic conditions in the southeastern United States (U.S.). Historically esteemed for its agronomic attributes, such as drought resistance, adaptability to acidic and nutrient-deficient soils, and ability to mitigate erosion via soil stabilization, SL is valued as a low-input forage crop for sustainable livestock production (Hoveland et al., 1990). Recently, focus on SL research and use by producers has shifted to its nutraceutical attributes for small-scale and resource-poor farmers (Terrill et al., 2012). Sericea lespedeza exhibits significant bioactivity against various economically significant internal parasites, including *Haemonchus contortus*, a blood-feeding gastrointestinal nematode of the abomasum, and *Eimeria* spp., protozoan parasites that cause coccidiosis in young ruminants (Shaik et al., 2006; Burke et al., 2013; Besharati et al., 2022). These parasites significantly contribute to mortality and production decline in small ruminants such as goats and sheep (Terrill et al., 2012; Kommuru et al., 2015). The anti-parasitic properties of SL have been attributed to a high concentration of condensed tannins consisting of over 90% prodelphinidin-type subunits (Kommuru et al., 2014; Machineni et al., 2014).

Aside from its anthelmintic properties, SL is associated with a host of additional advantages, such as decreased ruminal methane emissions (Naumann et al., 2013), inhibition of fly larvae in manure (Littlefield et al., 2011), and antimicrobial activity against specific bacterial pathogens (Min et al., 2008). This diverse range of benefits has led to a surge in demand for SL-based products, particularly seeds, hay, and pelleted leaf meal, among small ruminant farmers in the United States (T. Sims, Pers. Comm.). However, the crop’s diminutive seed size and sluggish initial growth present practical challenges for field establishment, necessitating innovative tools and technologies to enhance site selection and cultivation methods.

Agriculture, a fundamental sector of human civilization, is ever evolving due to changing environmental circumstances, technological advancements, and shifting socio-economic needs (Angelakis et al., 2020; Hemathilake and Gunathilake, 2022). A critical domain experiencing change is fodder management, particularly in resource-constrained environments. Emerging site-specific fodder management (SSFM) solutions are especially pertinent for farmers facing these limitations. Site-specific forage managment alows localized assessments of environmental variability and specific interventions to enhance forage production and sustainability (Antony et al., 2020; Dorji et al., 2022).

This transition is particularly relevant to SL production, where comprehending micro-level spatial changes in soil and climate is crucial for optimizing biomass yield and bioactive component concentration (Muir et al., 2014; 2017). Studies indicate that incorporating SSFM concepts into SL farming, particularly via precision agriculture techniques, can enhance the efficiency and sustainability of fodder production (Panda et al., 2020, 2023, 2023b).

Advocacy for use of SSFM has concentrated on underdeveloped nations; nonetheless, these measures are also pertinent in wealthier areas undergoing agricultural transformations. A slow transition from cattle to small ruminant farming has occurred in the southeastern U.S. due to diminishing land availability and climate unpredictability (Panda et al., 2023b). This tendency has accompanied a resurgence of interest in SL, whose cultivation has risen since the 1940s (Shaik et al., 2006; Panda et al., 2010).

In collaboration with local farmers, academics have developed decision support systems (DSS) to enhance SL management. These DSS platforms, leveraging geospatial technologies such as Geographic Information Systems (GIS), remote sensing, and Global Navigation Satellite Systems (GNSS), empower farmers with data regarding soil characteristics, topography, and climatic conditions. This geospatial framework facilitates preliminary biomass calculation, geographic forecasting of forage quality, and tailored management recommendations, essential elements of an effective SSFM-DSS model.

Notwithstanding its potential, the extensive use of GIS-based DSS in small-scale and sustainable agriculture is constrained by elevated data collection expenses, technical intricacies, and restricted capabilities for real-time analysis (Besim, 2023). Furthermore, these systems frequently exhibit insufficient reactivity to fluctuating variables, such as pest infestations or atypical weather occurrences, knowledge of which is essential for practical decision-making (Skendžić et al., 2021). Recent advancements have further reinforced the role of WebGIS in decision-making. For example, Recent bibliometric reviews highlight the increasing integration of artificial intelligence into decision support systems for smart agriculture, underscoring their potential in operational efficiency and sustainability (Yousaf et al., 2023). Rahman et al. (2023) demonstrated real-time WebGIS dashboards for rice farming in flood-prone areas, while Zhou et al., 2023 applied convolutional neural networks within a GIS interface for yield prediction in maize. These studies align with our work and emphasize the growing potential for integrating spatial intelligence with machine learning in DSS frameworks. Consequently, there is an urgent requirement for DSS platforms that are predictive, adaptive, and accessible to farmers with diverse technical skills.

Smartphone accessibility has contributed to narrowing this gap. Creating WebGIS dashboards combined with SSFM-DSS technologies is a viable option, facilitating real-time, location-specific insights for SL producers. This is especially pertinent for smallholder farmers in both developed and developing nations, where access to timely and actionable data can profoundly influence management results (Zhai et al., 2020).

The successful establishment of SL necessitates a deep understanding of its biological traits. The diminutive seed size and gradual initial growth require meticulous regulation of planting depth, soil density, and weed interference (Alexander et al., 2021). Furthermore, evaluating fodder quality is complicated. Traditional methods such as near-infrared reflectance spectroscopy (NIRS) may inadequately identify variations in condensed tannin levels, crucial for assessing SL’s anthelmintic and nutraceutical significance across diverse environmental and management contexts (Fraga-Corral et al., 2021; Stuth et al., 2003). This understanding is crucial for both researchers and farmers in determining the ideal conditions for maximizing biomass and bioactive chemical output. This heterogeneity poses difficulties for both researchers and farmers in determining the ideal conditions for maximizing biomass and bioactive chemical output of SL. Therefore, more advanced modeling techniques are required to evaluate the impact of environmental variables on SL’s growth and chemical profiles.

Artificial Neural Networks (ANNs) provide a robust machine learning methodology to tackle these difficulties. Modeled after biological neural networks, artificial neural networks (ANNs) have interconnected nodes, akin to neurons, that take inputs and undergo adaptation via a weight adjustment process referred to as training (Thakur and Konde, 2021). These models excel at identifying non-linear correlations among several variables, providing a significant advantage over conventional statistical methods (Abiodun et al., 2019). In agricultural research, ANNs have been effectively employed for yield prediction, disease identification, and resource optimization (Dahiphale et al., 2023). In SL production, ANN models can amalgamate geographical, meteorological, and biochemical facts to predict growth results and condensed tannin levels with enhanced precision. Traditional models such as multiple linear regression often assume linear relationships and can struggle with multicollinearity or variable interaction effects, which are common in agroecological systems. In contrast, ANNs can capture complex, nonlinear interactions without these assumptions, making them more suitable for modeling SL growth under diverse environmental conditions. Nonetheless, ANN models are frequently perceived as “black boxes” because of the obscurity surrounding their underlying decision-making mechanisms. This highlights the significance of their use of meticulous implementation, validation, and interpretability.

Despite these advances, there remains a clear gap in accessible, real-time decision support tools tailored for small-scale, resource-constrained farmers. This study directly addresses that gap by developing and validating a WebGIS-integrated SSFM-DSS specific to SL cultivation under variable environmental conditions. This project also utilizes ANNs to improve decision support systems for precise SL management. The model seeks to enhance the predictability and functionality of SSFM-DSS platforms by amalgamating geographical data with biochemical and environmental characteristics. These innovations facilitate sustainable forage production in the southeastern U.S. and offer a scalable model for fodder management. This research ultimately advances the overarching objective of creating resilient, technology-driven agricultural systems that harmonize productivity with ecological and economic sustainability.

## 2 Goals and objectives

This study was undertaken to develop a Site-Specific Fodder Management Decision Support System (SSFM-DSS) for *Lespedeza cuneata* (sericea lespedeza, SL). The ultimate goal of this system is to support the adoption of SL as a climate-resilient forage alternative in regions increasingly affected by flash droughts and environmental variability. The SSFM-DSS is significant as it provides a tailored approach to fodder management, taking into account the specific needs and conditions of SL, thereby enhancing its potential as a climate-resilient forage option (Christian et al., 2021). Previous research on SL has demonstrated exceptional adaptability to challenging conditions, including drought, acidic soils, and nutrient-poor environments, which often limit the sustainability of conventional forage crops (Hoveland et al., 1990; Panda et al., 2023b).

The current presented research applied a robust and adaptable methodological framework that combined field-based data collection, geospatial analysis, and predictive modeling. The first phase involved establishing standardized protocols for field sampling and laboratory analysis to ensure data consistency across locations and seasons. Key indicators, such as biomass yield, crude protein content, and condensed tannin concentration, were measured to assess forage productivity and anthelmintic potential. Ensuring procedural consistency was critical for model accuracy and for maintaining credibility with collaborating stakeholders (Odion et al., 2023).

Recognizing that environmental heterogeneity plays a decisive role in plant performance, a geospatial approach was employed to capture key spatial variables influencing SL growth. With this comprehensive dataset, we transitioned from descriptive analysis to predictive modeling. Both conventional statistical techniques and Artificial Neural Networks (ANNs) were used to model SL biomass production and nutritional profiles. ANNs, in particular, provide a powerful tool for capturing complex, non-linear interactions between variables, interactions that often elude traditional models. By evaluating key biophysical parameters such as soil drainage, pH, slope, and climatic extremes, the model produced actionable, high-resolution maps that identify optimal zones for SL cultivation. This tool transforms complex datasets into a practical decision-making resource, scalable across regions and accessible to a wide user base.

Altogether, this research represents more than a technical workflow. It is a step toward democratizing precision agriculture. By merging advanced geospatial analytics with ground truth data, this study delivers both scientific innovation and community-driven impact. The inclusion of farmers and stakeholders throughout the process ensures that the system is not only scientifically sound but also practical, inclusive, and poised for real-world application in climate-smart fodder production. The SSFM-DSS is designed to be user-friendly and accessible, ensuring that its benefits can be realized by a wide range of users.

## 3 Materials and methods

### 3.1 Rationale, scope, and agronomic advantage Of SL production

The development and yield of Sericea lespedeza (SL) are affected by a diverse interaction of environmental and soil conditions. Primary factors are precipitation patterns, temperature variations, particularly low temperatures, and soil attributes, like pH, texture, and fertility (Panda et al., 2023a). As established by previous research, growth of SL and specific plant attributes at the field level, such as plant height and stand density are affected by differences in terrain in different locations (Muir et al., 2014). Notwithstanding these dependencies, SL is proficiently adapted to flourish in severe environmental conditions, encompassing drought-prone areas and marginal soils characterized by low pH and restricted fertility (Hoveland et al., 1990). This versatility renders it a promising alternative for sustainable fodder production in regions increasingly impacted by changes in the environment.

From an agricultural point of view, SL is a fairly uniform crop once it is established. Weeds are usually the main cause of differences within a field. This consistency improves its use for remote sensing and precision agriculture purposes. Unique spectral fingerprints differentiating SL from adjacent weed species facilitate high-resolution picture classification (Siddique et al., 2024), resulting in precise yield estimation and effective weed detection. The ability to differentiate is a significant advantage for incorporating SL into geospatial monitoring systems that utilize satellite or UAV-derived images (Panda et al., 2023a).

Despite SL’s previous classification as an invasive species in some U.S. states, contemporary plant breeding initiatives have mitigated these issues. Enhanced non-invasive cultivars, including AU-Lotan (Donnelly, 1981) and AU-Grazer™ (Mosjidis, 2001), both produced at Auburn University, have reduced seed fracture and restricted dispersal, rendering them appropriate for controlled cultivation and expanded for commercial agricultural purposes. These cultivars preserve the plant’s fundamental agronomic advantages while mitigating environmental hazards, hence facilitating their integration into precision agricultural systems (Panda et al., 2020).

Besides its agronomic attributes, SL is renowned for its nutraceutical (nutritional + pharmaceutical) significance. The plant generates elevated concentrations of prodelphinidin-type condensed tannins, chemicals recognized for their antiparasitic efficacy, especially against *Haemonchus contortus* and *Eimeria* spp. in small ruminants (Kommuru et al., 2014; Mechineni et al., 2014). These tannins diminish parasite burdens without reliance on synthetic anthelmintics, providing a natural and sustainable alternative for animal health management.

Due to being in the leguminaceae family, SL enhances soil fertility by allowing biological nitrogen fixation, hence diminishing the necessity for nitrogen-based fertilizers. The extensive root system facilitates the absorption of nutrients such as phosphate and potassium from the subsoil (Horizon B), which are frequently unattainable for shallow-rooted forages and row crops (Hoveland et al., 1990). This renders SL not merely a low-input crop, but also a significant asset for enhancing soil structure and nutrient cycling in damaged fields.

Historically, SL has been employed in the U.S. for slope stabilization along roadways since the early 20th century, demonstrating its resilient root structure and extensive ground-covering growth habit. In the last ten years, research has focused on assessing SL as a premium feed for small ruminants. Fort Valley State University (FVSU) has been instrumental in this transformation, investigating the application of SL for sustainable livestock production in integrated crop-livestock systems throughout the southeastern United States (Silva et al., 2022; Walder, 2017).Recently, collaborative initiatives have broadened to encompass both smallholder and commercial-scale growers throughout the tri-state area of Alabama, Georgia, and South Carolina. These growers, in collaboration with researchers at FVSU, are examining SL as a sustainable alternative to conventional forages such as alfalfa (Medicago sativa), bermudagrass (Cynodon dactylon), and other grasses often grown for cattle. This switch, considering SL’s drought resilience, minimal input needs, and nutraceutical advantages, signifies a wider movement towards climate-smart forage options consistent with sustainable intensification concepts.

### 3.2 Study area

**Figure 1:**
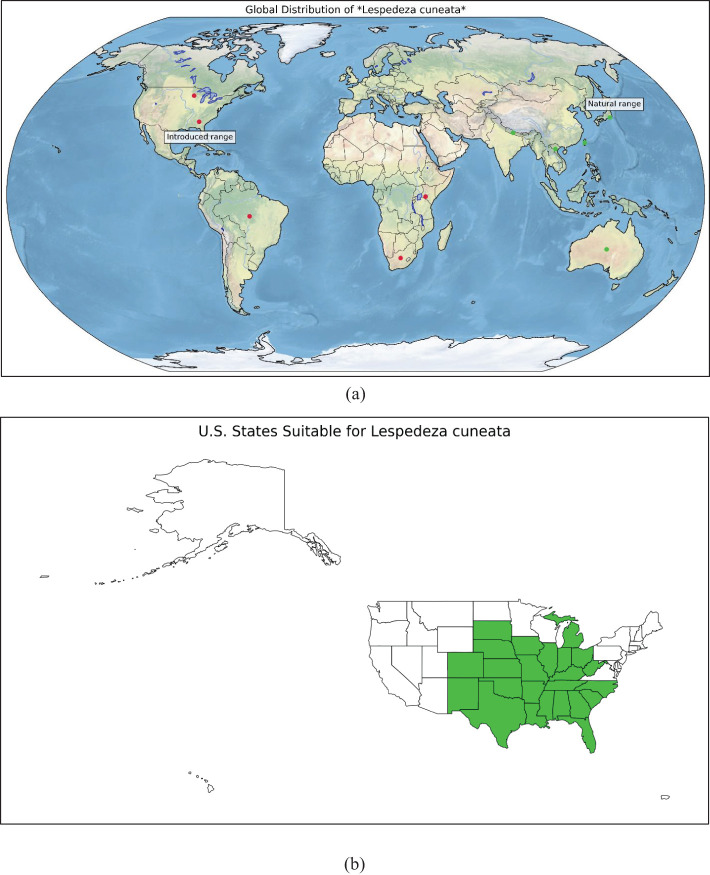
**(a)** Places of the world where sericea lespedeza is grown (Source: https://www.discoverlife.org/mp/20q?search=Lespedeza+cuneata) and **(b)** Places in the U.S. where SL is currently found (Source: University of Georgia - Center for Invasive Species and Ecosystem Health; Early Detection & Distribution Mapping System. Available online at https://www.eddmaps.org/; last accessed November 15, 2023).

**Figure 2:**
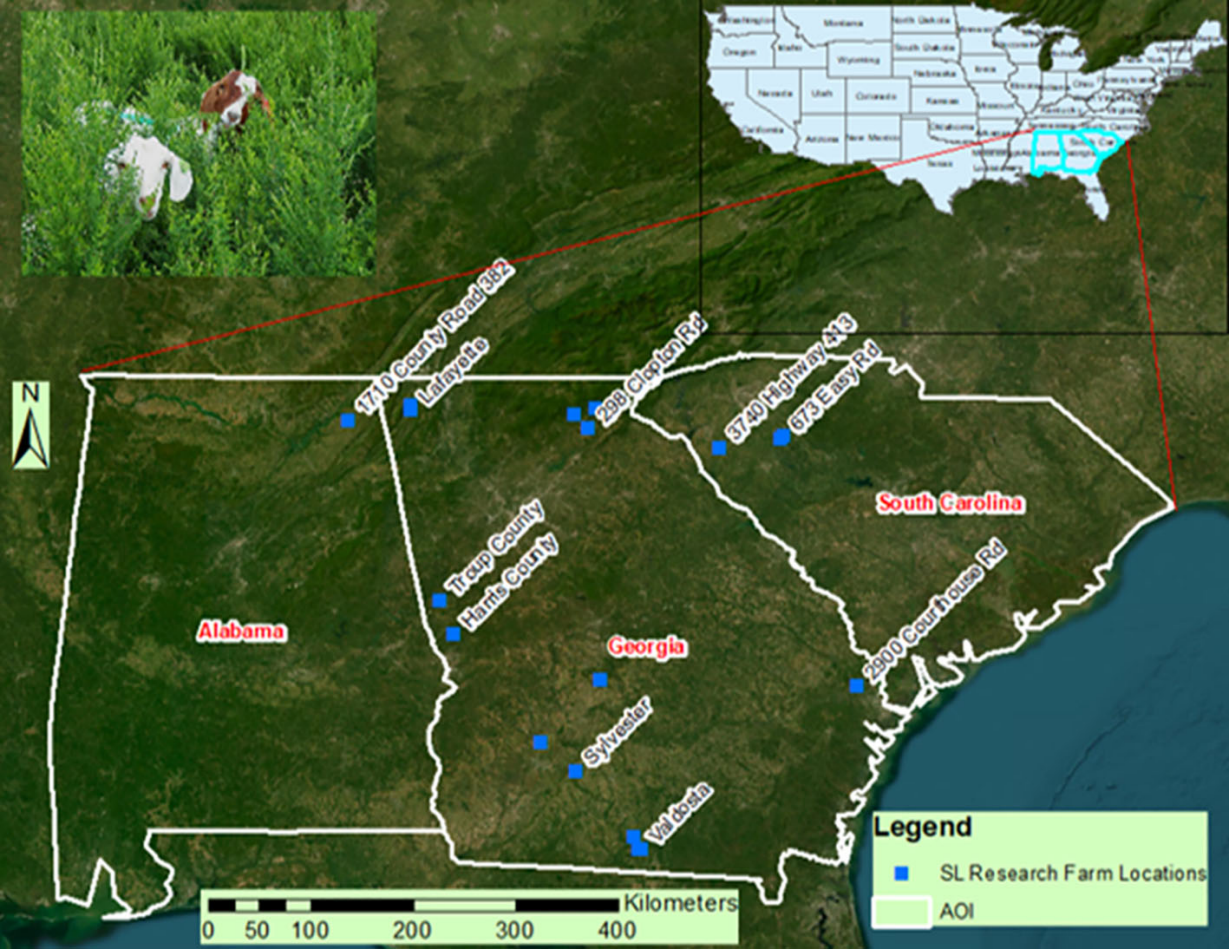
Twenty research sites in Alabama, Georgia, and South Carolina (from left to right) in the southeastern United States are marked by dots, representing where sericea lespedeza is currently grown. This includes a site at Fort Valley State University, GA, in the center.

**Figure 1a** illustrates the worldwide distribution of (sericea lespedeza, SL), whereas **Figure 1b** emphasizes the U.S. states with significant potential for extensive SL production, especially in the southeastern area. To facilitate the establishment of a geospatially up-to-date Site-Specific Fodder Management Decision Support System (SSFM-DSS) for SL, a comprehensive field study was executed across a Tri-State region, which includes Alabama, Georgia, and South Carolina (**Figure 2**), encompassing various agroecological zones in the southeastern U.S.

Twenty experimental sites were chosen throughout this region, including the Agricultural Research Station at Fort Valley State University (FVSU), at 32.5383° N, 83.8910° W. The study area extended from approximately 30.3° to 35.0° N latitude and 81.0° to 88.5° W longitude, incorporating a diverse array of environmental gradients related to climate, elevation, and soil conditions. These 20 sites were farmers-owned field, where they grow SL, and our research team studies the SL growth with contributing agronomic and environmental parameters. These sites are situated in heterogenous environmental conditions, such as differing topography and ecoregions in three different southeastern states, Alabama, Georgia, and South Carolina, ranging from mountainous sloped regions on the Appalachian foothills, moderately sloped piedmont clay areas, and the gulf coastal flat lands. These environmentally and agronomically heterogeneous sites with actual farmers management (no controlled management practices) makes our research model and its results robust. The involvement of farmers was pivotal to this research design. Their involvement not only enhanced the practical relevance of the findings but also fostered a collaborative approach to research in the agricultural sector.

The agroecological variability of the Tri-State region provided an optimal natural laboratory for assessing SL performance under diverse settings. Alabama’s agroecological zones extend from the Appalachian Plateau in the north, a region known for its unique soil and climate conditions, to the Gulf Coastal Plain in the south, a region with distinct soil and climate characteristics. Georgia encompasses the Blue Ridge Mountains, Piedmont, and Coastal Plain, transitioning from acidic clay soils and undulating hills to level, lush, sandy loam-rich agricultural plains. South Carolina encompasses mountainous regions in the Blue Ridge Escarpment and coastal alluvial plains, exhibiting diverse rainfall and temperature trends throughout these areas.

Topographic variance was significant throughout the research sites. In the Appalachian Mountain foothills, the northern regions of Alabama and Georgia attained altitudes beyond 1,000 meters above sea level, whilst coastal areas in southern Georgia and South Carolina typically measured only a few meters above sea level. This gradient influenced microclimatic factors, like temperature, precipitation patterns, and frost-free days. Northern locations encountered colder weather with increased yearly precipitation, whilst southern locations displayed warmer, arid conditions and were more susceptible to drought, which was a critical consideration in assessing SL’s climate resilience.

The soil types at the sites comprised Ultisols, characteristic of the Piedmont region, with poor natural fertility and high acidity; Alfisols in transitional zones; and Entisols and Inceptisols along the coastal plain, with elevated organic matter in certain regions. The soils exhibited pH, texture, and moisture retention variations, creating an extensive testing environment for SL’s adaptability. Environmental heterogeneity was essential for developing prediction models, enabling machine learning algorithms to understand how variations in site circumstances influence SL growth and nutritional quality.

The work produced a comprehensive dataset for training and validating the SSFM-DSS. By integrating various agroecological, meteorological, and edaphic factors, this architecture allows for site-specific recommendations. However, the model outputs are not confined to the study area only, but have the potential to be applied to analogous contexts beyond the study area, offering a broader applicability and a promising outlook for the potential impact of the study.

### 3.3 Data collection and processing

#### 3.3.1 Agronomic importance geospatial data collection

To improve the accuracy and contextual depth of our research, a collection of geographical and environmental datasets was methodically incorporated into the analytical framework. This integrative approach facilitated a more thorough comprehension of landscape-level heterogeneity and its impact on study results.

The 2021 version of the National Land Cover Database (NLCD; https://www.mrlc.gov/data) was key to the geographical analysis. This dataset, meticulously constructed through comprehensive classification and corroborated with ground-truth observations, provided a dependable basis for analyzing land cover trends. The NLCD data facilitated identification and quantification of vegetation types and land use categories within the Area of Interest (AOI), hence permitting stratified sampling and habitat classification.

A 30-meter resolution Digital Elevation Model (DEM) was acquired from the USDA-NRCS Geospatial Data Gateway (https://datagateway.nrcs.usda.gov/) to enhance the understanding of spatial heterogeneity. The DEM enabled topographic analysis, encompassing slope, aspect, elevation gradients, which are essential factors in ecological modeling and land suitability evaluations. This dataset was specifically utilized to estimate microclimatic variables and drainage patterns that could affect vegetation health and pest dynamics.

Soil characteristics were integrated using a dual methodology involving the State Soil Geographic (STATSGO) and the Gridded Soil Survey Geographic Database (gSSURGO). The STATSGO offered a comprehensive overview of soil types and their distribution at the state level, which was beneficial for regional planning and suitability modeling. Conversely, the gSSURGO database provided superior spatial resolution and comprehensive soil property data (e.g., texture, organic matter content, pH) for accurate, field-level studies crucial for comprehending crop-environment interactions.

High-resolution aerial images obtained from Unmanned Aerial Vehicles (UAVs) were essential for improving spatial data precision. The UAV surveys facilitated real-time, high-definition assessment of vegetation structure, canopy coverage, and alterations in land use within the AOI. The drone-based observations were crucial for validating land cover classifications and identifying minor spatial patterns that would be overlooked in lower-resolution datasets. In regions with restricted UAV access, due to legislative, logistical, or physical limitations, satellite imagery from the Landsat program was utilized. The integration of Landsat data guaranteed temporal continuity and spatial comprehensiveness throughout the study area. While UAV imagery offered high spatial resolution and flexibility for site-specific monitoring, it was sometimes constrained by battery life, limited flight range, and unfavorable weather conditions. In contrast, Landsat imagery provided broader spatial and temporal coverage but had lower resolution and was occasionally affected by cloud cover or timing mismatches with field sampling dates. These factors introduced challenges in aligning remote imagery with on-ground observations. Additionally, processing both UAV and Landsat imagery required advanced technical skills and specialized software, which could limit their accessibility for smallholder applications.

Climate data were obtained from the PRISM Climate Group at Oregon State University to integrate temporal variability and address evolving environmental circumstances. The datasets comprised high-resolution, daily records of precipitation, soil moisture, and temperature extremes (minimum and maximum), essential for modeling dynamic environmental impacts on the variables of interest. The incorporation of real-time and historical climate data facilitated a timely analysis, identifying seasonal trends and specific weather events that could have influenced biological or ecological processes in the study. The integration of this varied information created a strong, multidimensional basis for spatial and environmental modeling, enabling the study to tackle biophysical complexity and spatial variability with enhanced precision.

#### 3.3.2 Instrumentation and methodology for field sample collection

**Figure 3:**
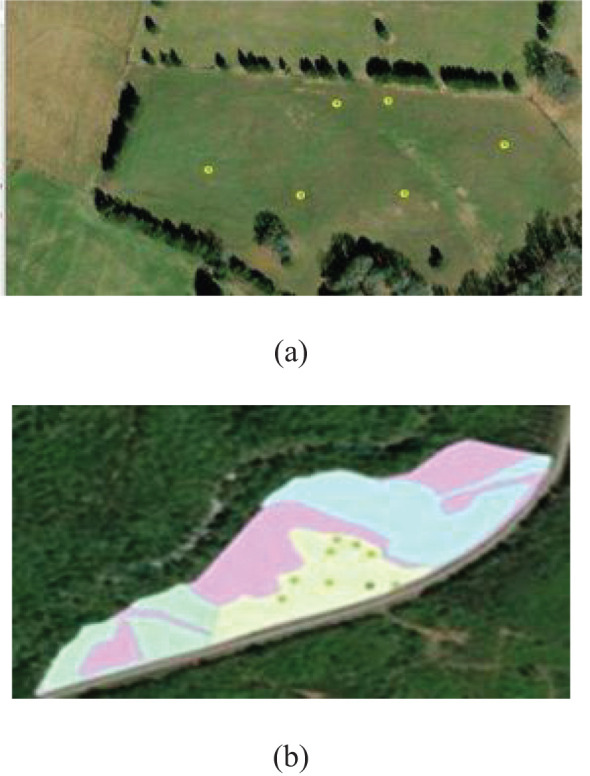
Representation of the stratified random sampling for GPS points on sites of sericea lespedeza production **(a)** over UAV collected and processed image and **(b)** gSSURGO soil spatial distribution superimposed site map.

Stratified random sampling was used to ensure that each sub-region within the tristate area, characterized by distinct agroecological features, was proportionally represented. The area was divided into strata based on elevation, slope, and land cover classes derived from Landsat imagery. From each stratum, random sampling points were generated and adjusted based on accessibility and field conditions. The region was stratified into classes based on elevation (low, mid, high), slope gradient (gentle, moderate, steep), and dominant land cover types (e.g., cropland, pasture, forest). Within each stratum, a set number of sampling points were randomly selected. If a selected point was inaccessible, the nearest accessible location within the same stratum was chosen to preserve stratified representation. Field-level sampling was performed to evaluate the biomass yield and fodder quality of SL, alongside the collection of geographical data with agronomic relevance. A uniform sample technique was utilized with a 3.66-meter rope, positioned at various locations within each field, to define circular plots, where all vegetation was trimmed to a stubble height of 5 cm. This method guaranteed consistency in sample collection and enabled precise biomass assessment. Sampling sites were chosen using a stratified random sampling methodology to account for within-field variability, as depicted in **Figures 3a, b**. **Figure 3a** illustrates the sampling locations (yellow dots) superimposed on a drone image of a typical field site, whereas **Figure 3b** presents the sampling spots in conjunction with a soil classification map obtained from the gSSURGO dataset for a different site.

Depending on field heterogeneity, five to eight sampling locations were chosen for each field. The exact geographic coordinates of each location were documented utilizing a Global Positioning System (GPS) device (Garmin GPSMAP 64, Part number 010-01199-00, Olathe, Kansas), facilitating spatial referencing and integration with remote sensing data. The SL biomass gathered at each site was deemed indicative of the corresponding field, facilitating *in situ* assessment of both fodder quantity and quality, which were subsequently examined concerning the previously described spatial agronomic characteristics.

At each sampling location, the height of three randomly chosen SL plants was measured and averaged to provide as an indicator of vertical biomass distribution. Despite the majority of the aboveground biomass being removed, a residual height of 5 cm was intentionally preserved to promote regrowth. The collected biomass was promptly weighed in the field with a hand-held digital scale (AvaWeigh HSD330, PA, USA), followed by the extraction of subsamples for laboratory analysis. The subsamples were frozen, freeze-dried, and milled to a particle size of 1 mm using a Cyclotec Sample Mill (FOSS CT293, Denmark). Forage quality was evaluated via laboratory analysis to measure nutritional value components, including extractable condensed tannins (ECT, %), protein-bound condensed tannins (PBCT, %), fiber-bound condensed tannins (FBCT, %), and total condensed tannins (TCT, %).

The aggregate weight of fodder harvested (in kg) was documented and evaluated to assess biomass yield per plot. A supplementary metric—biomass volume—was computed by multiplying the mean plant height by the area of the sampling circle, determined as Πr², where r denotes the radius established by the 3.66-meter rope. An allometric indicator denoting the canopy-to-stem area ratio is in development and will be tested using LiDAR-derived structural vegetation data, enhancing the comprehension of plant architecture and biomass distribution.

### 3.4 Geospatial data acquisition and processing for fodder quality and quantity remote estimation

The study employed a systematic, two-tiered approach to collecting and processing geospatial data, aimed at integrating field-collected information with remote sensing and spatial databases. Geographical coordinates were obtained in the field with portable GPS devices while sampling. The coordinates were transformed into a Point Shapefile format, incorporating latitude and longitude attributes, a conventional method for geographical representation and analysis in geographic information systems (GIS).

The boundaries of the SL cultivation fields were digitized using high-resolution (1-meter) National Agriculture data Program (NAIP) data as a foundation map to clearly define the areas under research (**Figures 3a, b**). This method facilitated a precise spatial delineation of the sampling regions. Simultaneously, UAV-acquired footage was gathered from most field locations. When UAV imagery was inaccessible, Landsat 8 satellite data was utilized to maintain spatial coverage continuity. Landsat 8 scenes were meticulously chosen to align closely with the actual field sampling dates, eliminating temporal data interpretation discrepancies. The satellite data were obtained from the Earth Explorer platform operated by the U.S. Geological Survey (USGS) (https://earthexplorer.usgs.gov/).

Supplementary raster layers were aggregated to enhance the geographic dataset, comprising digital elevation models (DEMs), soil classification data, and meteorological variables, including precipitation, temperature (minimum, maximum, and average), sun radiation, and land cover. The selected rasters, intended to offer an extensive overview of the research areas, were standardized to a spatial resolution of 30 meters and projected using the Universal Transverse Mercator (UTM) system with the North American Datum 1983 (NAD83). All datasets were constrained to the defined borders of the 20 study sites to ensure congruence with the Landsat images.

Additionally, Color Infrared (CIR) images—comprising Red, Green, and Near-Infrared (NIR) bands—and Thermal Infrared (TIR) imagery obtained by UAVs at certain locations were processed and confined to sample regions having a circumference of 609.60 cm. The high-resolution data products, representing the forefront of geospatial science, facilitated the creation of agronomic raster layers that aided in developing an automated geospatial model. This approach was developed to facilitate spatial decision-making for SL production by assessing site-specific appropriateness.

A prediction method for evaluating SL fodder quality and biomass was constructed using various image-based and environmental information layers. The analysis encompassed spectral indices, including the Normalized Difference Vegetation Index (NDVI) and Soil Adjusted Vegetation Index (SAVI), distinct spectral bands (Red, Green, NIR, and TIR), as well as site-specific environmental variables, such as elevation (measured *in situ* via GPS), precipitation, temperature metrics, and solar radiation.

**Figure 4:**
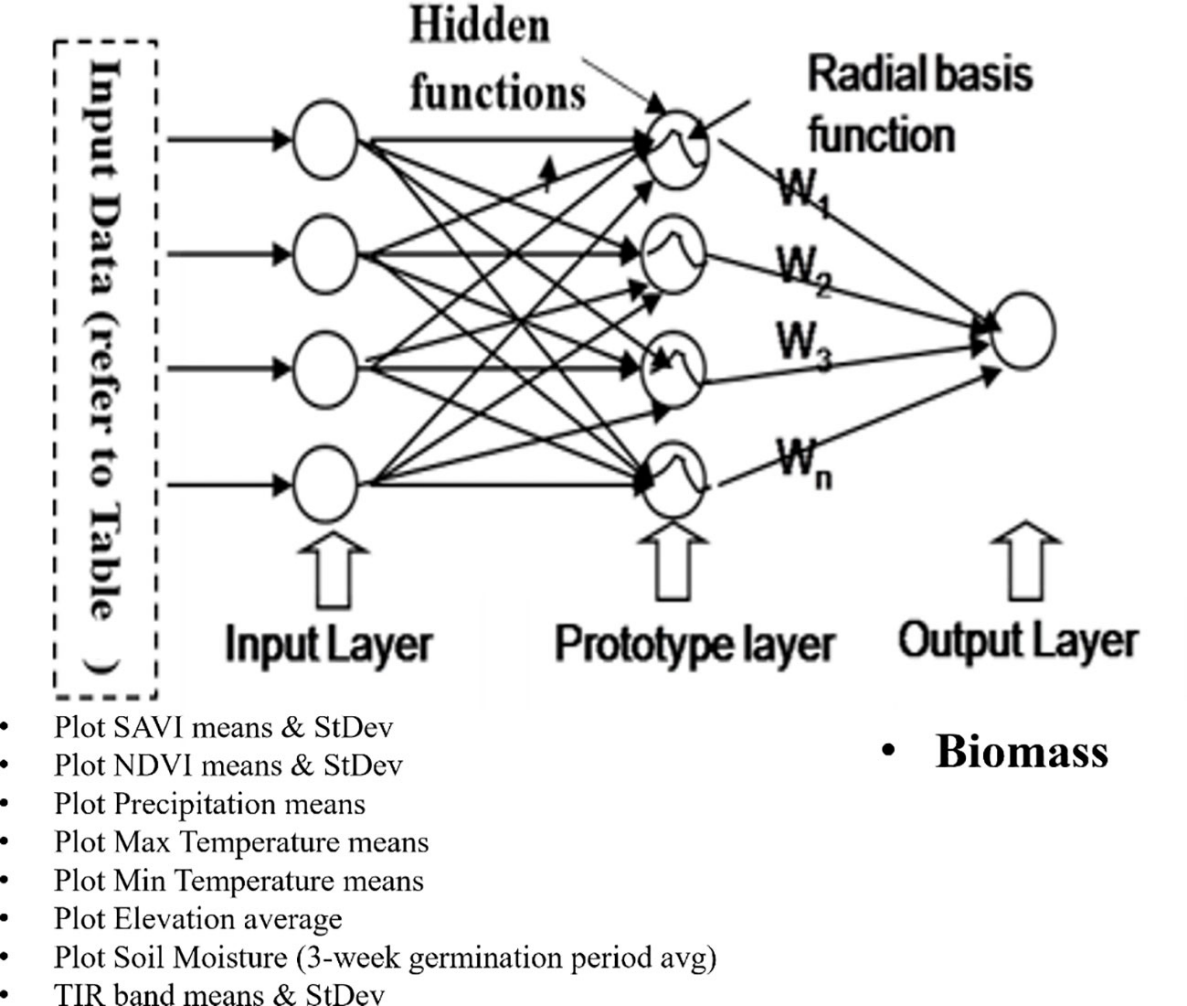
RBFN model architecture to estimate variations in sericea lespedeza crop quality and quantity parameters using ANN software.

**Table 1** delineates the input and output parameters employed in model construction. A multiple regression analysis was conducted with the MS-Excel Statistical Data Pack, and a Radial Basis Function Network (RBFN) model was developed using NeuralWare Professional II Plus software. **Figure 4** depicts the architecture of the RBFN model, emphasizing the configuration of input neurons and a singular output neuron (e.g., biomass) to exemplify the predictive modeling process for each dependent variable.

**Table 1:** Table showing database developed to estimate variations in SL crop quality and quantity parameters, using MS-Excel Statistics Tool Pack.

Farm	Input parameters											Output parameters					
	NDVI Mean	NDVI Std	SAVI Mean	SAVI Std	Elevation Mean (m)	Elevation Std (m)	Avg. Precipitation (mm)	Min Temp (^0^C)	Min Temp (^0^C)	Band10 Mean (16-bit)	Band10 Std (16-bit)	ECT %	PBT %	FBT %	TCT %	mg Binding CT/g plant	Biomass (lb)
AL1	0.29	0.03	0.53	0.05	248.20	2.13	1414.02	8.58	20.21	43,259.00	224.65	1.17	1.59	0.00	2.76	4.11	25.01
GA1	0.23	0.02	0.41	0.45	158.11	2.83	1142.61	9.61	23.26	43,141.69	565.14	7.94	2.92	0.00	10.86	62.64	19.05
GA2	0.37	0.03	0.67	0.06	48.20	1.00	1256.79	13.16	25.88	46,994.49	473.31	0.47	3.58	0.00	4.05	15.00	16.27
GA3	0.30	0.03	0.54	0.06	108.72	2.73	1168.40	11.74	24.70	47,909.33	364.40	1.77	3.06	0.00	4.83	6.30	25.50
GA4	0.33	0.02	0.59	0.04	115.71	0.45	1168.40	11.74	24.70	47,857.13	323.28	4.00	6.69	0.01	10.69	16.59	15.46
GA5	0.31	0.03	0.56	0.05	103.96	4.37	1219.20	12.79	25.28	47,032.92	249.50	4.00	6.69	0.01	10.69	16.59	14.55
GA6	0.48	0.03	0.86	0.07	539.48	2.38	1625.60	7.37	19.99	47,000.00	248.00	1.27	3.23	0.00	4.50	1.48	5.55
GA7	0.29	0.05	0.53	0.09	278.94	4.23	1320.80	9.03	21.32	43,364.72	261.60	2.19	2.59	0.00	4.78	7.00	16.83
GA8	0.29	0.03	0.53	0.05	248.20	2.13	1414.02	8.58	20.21	43,259.00	224.65	3.33	3.63	0.00	6.95	16.03	3.78
GA9	0.29	0.05	0.53	0.09	278.94	4.23	1320.80	9.03	21.32	43,364.72	261.60	4.19	2.50	0.00	6.69	26.10	16.34
SC1	0.38	0.03	0.69	0.06	174.73	7.01	1162.30	9.70	22.67	43,455.45	130.66	8.99	3.80	0.00	12.79	95.17	14.13
SC2	0.25	0.02	0.45	0.03	193.82	2.25	1143.00	9.29	22.85	44,964.82	343.21	8.13	3.25	0.00	11.38	72.08	15.64
SC3	0.29	0.03	0.53	0.05	411.52	0.79	1625.60	7.83	20.90	43,826.65	231.13	2.90	3.79	0.00	6.69	19.70	8.80

NDVI, Normalized Difference Vegetation Index; SAVI, Soil Adjusted Vegetation Index; Band10, Thermal Infrared Band 10 (Landsat 8); ECT, Extractable Condensed Tannins; PBT, Protein-Bound Tannins; FBT, Fiber-Bound Tannins; TCT, Total Condensed Tannins; mg Binding CT/g plant, milligrams of condensed tannin binding capacity per gram of plant material.

### 3.5 Automated SL production spatial suitability determination model in the tristate

The geospatial datasets outlined in Section 3.4 were obtained and analyzed to facilitate the creation of a spatial suitability model for specific legume production in the Tristate study area. This modeling initiative integrated essential soil parameters that affect crop productivity, such as soil loss tolerance (indicative of stability), drainage class (reflecting moisture dynamics), soil texture (linked to fertility and nutrient retention), available water content (AWC), saturated hydraulic conductivity (kSat), and soil pH. Each parameter influences the regional heterogeneity of crop performance by facilitating the interactions between soil characteristics and plant growth.

The variables were transformed into raster layers utilizing the Reclassify tool in ArcGIS Pro (ESRI, Redlands, CA) to enable spatial analysis. The reclassification technique normalized all variables on a suitability scale ranging from 1 (least suited) to 5 (most suitable). This transformation adhered to a Delphi Scaled Value Determination process (Panda et al., 2023), wherein expert input was utilized to allocate relative appropriateness scores according to the agronomic significance of each parameter. The Delphi process involved five experts specializing in agronomy (TT), soil science (AKM), remote sensing (SSP), and forage systems (TT). Each expert independently reviewed the thirteen environmental parameters and assigned a suitability score (1–5) based on the parameter’s impact on SL growth. Scores were then aggregated, and discrepancies discussed in a follow-up round to reach consensus. This structured and iterative approach ensured that the final weights reflected both empirical knowledge and practical experience relevant to SL cultivation in the southeastern U.S.

The Delphi approach utilizes organized expert input to evaluate the comparative significance of various aspects in a decision-making framework. The involvement of domain specialists in this procedure guaranteed that the designated values accurately represented real field experience and regional crop necessities.

Simultaneously, topographic variation was examined by generating a slope raster from the digital elevation model (DEM) of the Area of Interest (AOI) utilizing the Slope tool in ArcGIS. This layer was classed based on the 1–5 appropriateness scale. All classification rasters, including soil, slope, and other environmental factors, were standardized to conform to the format of the gSSURGO-derived raster data, facilitating seamless integration into the comprehensive suitability model.

**Figure 5:**
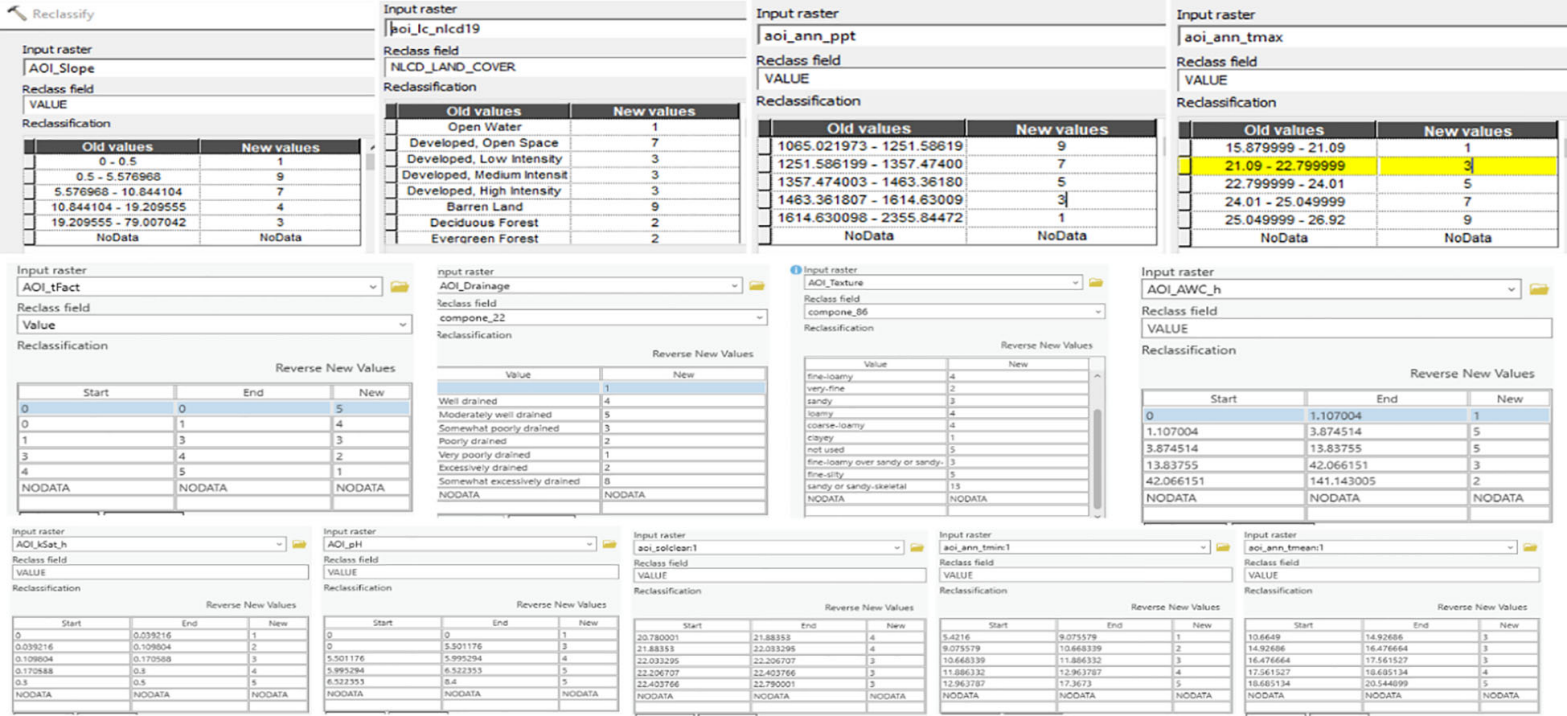
The Delphi-based sericea lespedeza production suitability scale values on a scale of 1 (Least Suitable) and 5 (Most Suitable) of all 13 rasters, with each parameter shown in its corresponding Input Raster Text Box.

**Figure 5** depicts the geoprocessing workflow created in ArcGIS Pro (version 3.2.2) utilizing the ModelBuilder platform. This automated model delineates the entire sequence of processes, encompassing raster reclassification and integration phases, organized as a coherent flowchart. The final model result was a composite raster, the SL Production Spatial Suitability Raster, generated by amalgamating all 13 classed layers.

The integration process utilized a weighted overlay method informed by the Delphi technique to allocate relative significance to each input parameter. Weights of 5% were assigned to each of the seven soil-based factors, as well as to the maximum and average temperature layers. The minimum temperature and precipitation, deemed to have a greater impact on SL crop performance, were each allocated a weight of 10%. The land use/land cover (LULC) layer was assigned the highest weight of 15%, recognizing its essential function in assessing land availability and agricultural potential.

**Figure 6:**
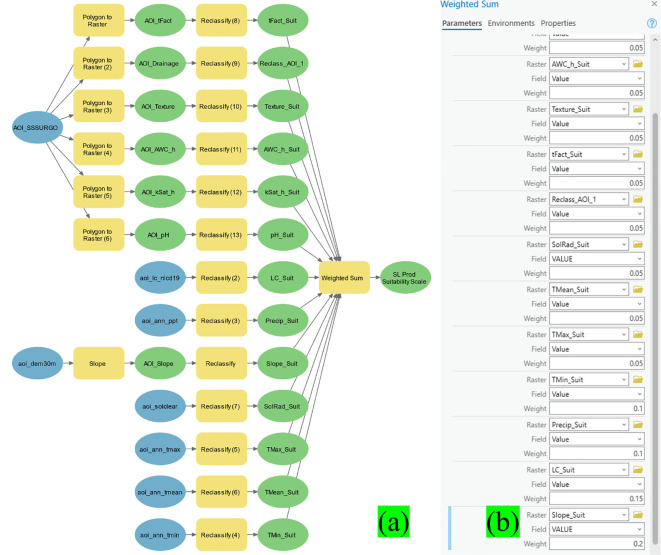
Automated sericea lespedeza production suitability determination model **(a)** with the Weighted Sum process weighted scores shown in decimals **(b)**.

**Figure 6** displays the 13 input rasters together with their corresponding Suitability Scale Values utilized in the reclassification and weighted integration procedure. The spatial suitability model offers a comprehensive, expert-guided framework for pinpointing high-potential zones for SL cultivation in the Tristate region, facilitating informed decision-making in agricultural planning and land use management.

### 3.6 WebGIS dashboard development for SSFM-DSS dissemination

According to Fleming et al. (2022), ArcGIS Dashboards are robust instruments for obtaining and analyzing intricate geospatial data. They are intuitive for users and integrate geographical data with user decision-making. This integration allows farmers and other agricultural stakeholders to observe occurrences, discern trends, and make informed decisions based on real-time geographic information. The dashboard, tailored to cultivating Sericea lespedeza (SL), allows users to effortlessly select a geographic point of interest to evaluate the suitability of a given place for SL production based on combined agronomic and environmental parameters.

If a chosen site is considered unsuitable for SL production, the dashboard notifies users of the agronomic aspects requiring modification, such as soil characteristics, pH levels, or meteorological conditions, to enhance appropriateness. This degree of interaction not only improves accessibility but also plays a crucial role in facilitating precision agriculture. By customizing recommendations to site-specific conditions, the dashboard significantly enhances its value for the audience, making it an indispensable tool for agricultural decision-making.

ArcGIS Dashboards are engineered to display numerous interrelated visualizations within a single interface. For example, selecting a map point can dynamically display windows containing attribute tables or model results, enhancing layered data discovery. The WebGIS Dashboard utilized in our project functions as a comprehensive spatial decision support system (SSFM-DSS) for SL production throughout the Tri-State area, ensuring that all aspects of the production process are covered. It compiles essential insights into an accessible web map style, transforming intricate datasets into practical information for users.

The completed online dashboard features a project summary, a well-defined model schematic, and the conclusive spatial suitability data for SL production throughout the region (see **Figure 6**). Collectively, these elements facilitate swift, evidence-driven decision-making for sustainable land utilization and enhanced forage crop production. While the current study utilized data from 20 strategically chosen experimental sites, this sample size may limit the generalizability of the SSFM-DSS model across the entire tristate region. Future studies should aim to validate the model using expanded datasets from other geographic zones, soil types, and environmental conditions to improve its robustness and scalability for wider adoption.

## 4 Results and discussion

### 4.1 SL quality and quantity remote estimation

A spatially explicit assessment was conducted to determine the suitability of sericea lespedeza (SL) cultivation across the southeastern United States, focusing on Georgia, Alabama, and South Carolina. To capture the multifaceted environmental requirements of SL, the modeling approach incorporated 13 critical biophysical variables, including soil drainage, texture, pH, slope, precipitation, and others known to impact plant establishment and productivity. Each variable was converted into standardized raster layers, reclassified into a common suitability scale, and synthesized using ArcGIS ModelBuilder. The Jenks natural breaks algorithm was employed to classify overall suitability scores, ranging from 1 (least suitable) to 5 (most suitable), providing a nuanced composite map (Panda et al., 2023b).

**Figure 7:**
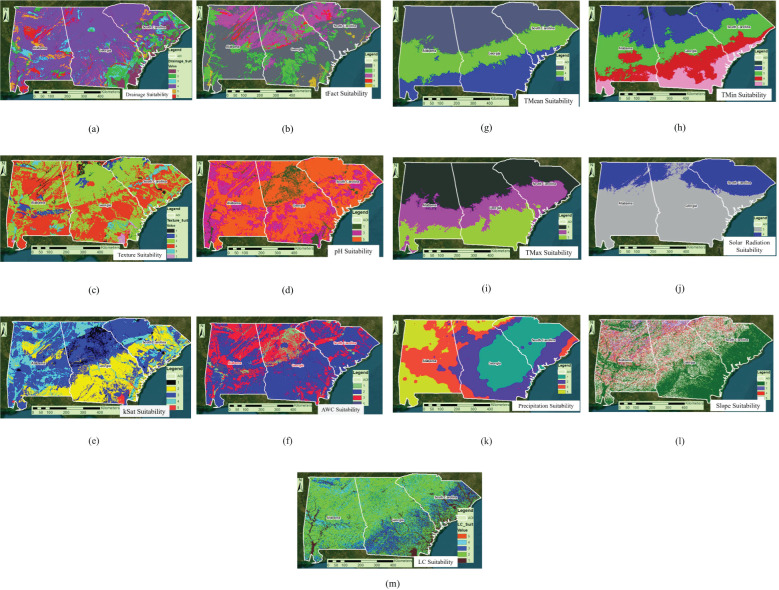
**(a-m)** Thirteen sericea lespedeza production agronomic factor suitability-scaled rasters through a scale of 1-5, with 1 indicating the least suitable, 2 = lowly suitable, 3 = moderately suitable, 4 = highly suitable, and 5 = most suitable.

**Figures 7a–m** illustrate the spatial distribution of each environmental factor and its respective influence on SL suitability across the tri-state region. For instance, **Figure 7a** emphasizes the significance of soil drainage. Moderately drained soil, offering balanced water retention and aeration were rated highly, as they promote healthy root development and reduce risks associated with drought or waterlogging. Conversely, excessively drained or poorly drained soils received lower scores due to extremes in water availability. Similarly, **Figure 7b** shows that highly erodible soils negatively affect SL suitability, as erosion can compromise long-term plant anchorage and reduce soil fertility. In **Figure 7c**, soil texture further clarifies suitability, with clay-heavy soils scoring poorly due to compaction, poor aeration, and limited water infiltration, consistent with field observations in northeast Georgia.

Soil pH, depicted in **Figure 7d**, is another pivotal factor. SL preferred slightly acidic soils (pH 5.5–6.5), corroborating prior studies in Eswatini and other regions (Panda et al., 2023). Hydrological variables further influenced suitability, including saturated hydraulic conductivity (kSat) and available water capacity (**Figures 7e, f**). Extremes in either direction reduced scores due to excessive permeability or poor drainage capacity. Climatic variables also played a critical role. **Figure 7h** highlights minimum temperature as a limiting factor for SL establishment, particularly in higher-elevation or northern zones. **Figures 7j, k** show that both low solar radiation and excessive precipitation can create suboptimal conditions by promoting evapotranspiration stress or water saturation, respectively. Topographic and land cover factors added further nuance. As shown in **Figures 7l, m**, pasturelands and gently sloping terrains (1–5%) were the most conducive to SL growth, offering ease of mechanization, moderate runoff, and suitable habitat conditions. Wetlands and forested areas, by contrast, were deemed unsuitable due to competition, shading, and potential flooding. Although real-time data integration remains a challenge, future versions of the SSFM-DSS could incorporate IoT-based sensors, satellite telemetry, and mobile app interfaces to automatically capture field-level conditions such as soil moisture, temperature, and biomass indicators. Establishing secure data pipelines and using cloud-based geospatial platforms may also enhance real-time feedback and improve the system’s decision-making accuracy.

**Figure 8:**
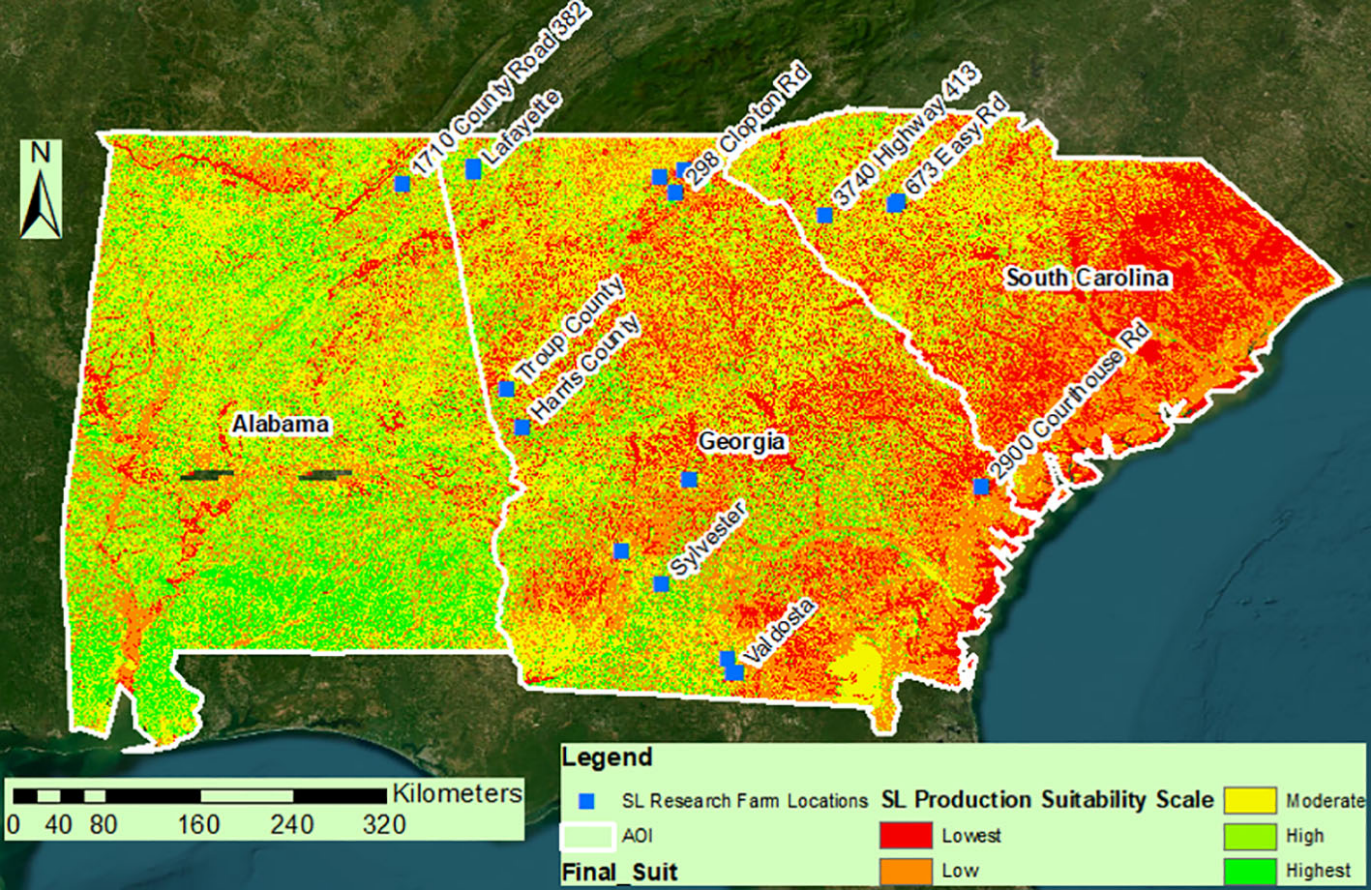
Study resultant map showing the spatial suitability of SL production in a scale of 1-5 (lowest to Highest suitability) as generated using Jenks algorithm while classifying the raster.

The final composite suitability map (**Figure 8**) effectively integrates all these factors, revealing distinct high-potential zones. Unsuitable areas are often aligned with intensive row-crop zones or coastal regions prone to salinity or drainage issues. In contrast, herbaceous and shrubland zones, frequently underutilized, emerged as prime candidates for SL cultivation. This information can guide land managers in making informed decisions about land use and crop selection. Importantly, all 20 on-farm trial sites fell within areas rated as Class 4 or 5, validating the predictive strength of the model. This outcome aligns with similar site-specific modeling efforts in viticulture (Barbetti et al., 2024), illustrating how environmental zoning can support precision forage management.

### 4.2 Integrating remote sensing for biomass estimation

Remote sensing data were combined with ground-truthed observations from 41 georeferenced plots to estimate SL biomass in the field. Spectral indices: Normalized Difference Vegetation Index (NDVI) and Soil Adjusted Vegetation Index (SAVI) were paired with elevation and temperature variables to model biomass production. A multivariate linear regression model accounted for 54% of the variance (R² = 0.54), with a standard error of 0.736 kg, indicating a moderate level of predictive power. These findings are consistent with other studies in water-limited systems (Zhao et al., 2022), where vegetative indices are influenced by environmental stress. To enhance model performance and account for non-linear interactions, we implemented a Radial Basis Function Network (RBFN) a type of Artificial Neural Network (ANN). The RBFN model demonstrated substantial predictive improvement, achieving 86% accuracy on the training and 80.5% on the validation set. The training and testing RBFN modeling RMSE obtained were 0.63 and 0.59, respectively. The standard error of prediction (SEP) of RBFN training and testing models were 0.58 lb (3.6%) and 0.55 (3.4%) of biomass yield. This jump in performance underscores the value of ANN models for capturing complex biophysical relationships that traditional linear models may miss. These results echo findings from Kim and Kisekka (2021), who highlighted the superior adaptability of machine learning models in precision agriculture. This modeling approach underscores the practicality of combining remote sensing with advanced analytics for biomass forecasting, especially in regions where field measurements are resource-constrained or time-consuming. This adaptability empowers users in such regions to make informed decisions without the need for extensive resources.

### 4.3 Advancing WebGIS decision tools

**Figure 9:**
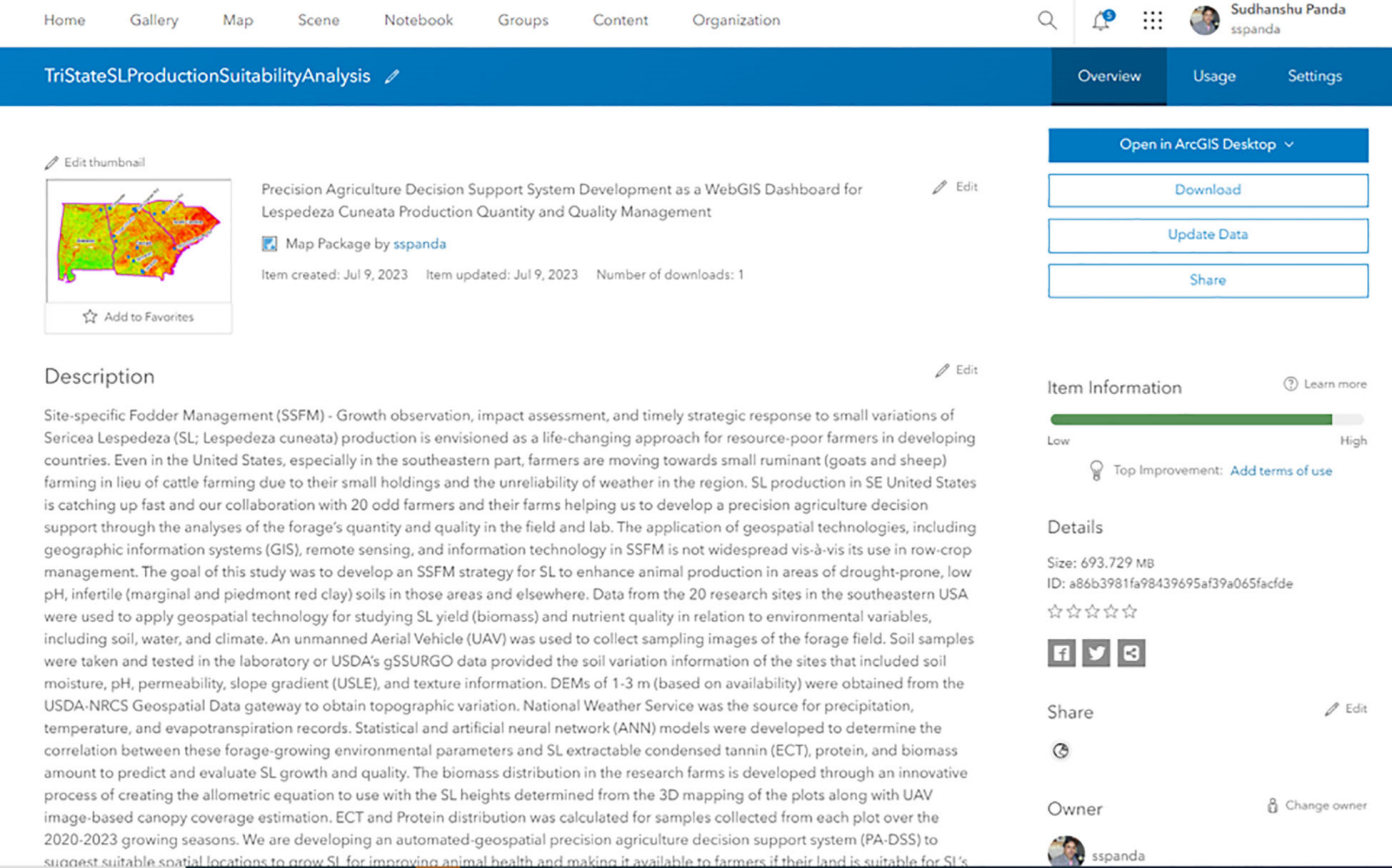
A screenshot for the decision support map showing Tristate SL production suitability in ArcGIS Dashboard for easy access by end-users.

To translate spatial data into actionable insights, we developed a WebGIS dashboard (**Figure 9**) using ArcGIS. This interactive tool enables a range of stakeholders including farmers, extension agents, and land managers to access SL suitability and biomass estimates in real time. The dashboard can provide valuable information for decision-making, such as where to establish new SL fields or how to optimize existing ones. Users can select specific locations, view environmental suitability layers, and receive location-based recommendations, enhancing their ability to make informed decisions. The dashboard serves as a user-centric decision support platform, similar in concept to tools like WaterSmart-GIS (Zhao et al., 2022) and GoProsit (Barbetti et al., 2024). Incorporating interactive mapping, data overlays, and simplified visual cues bridges the gap between complex spatial analysis and practical on-farm decision-making. Importantly, the tool’s interface is designed to be intuitive for non-specialists, enhancing its utility in agricultural outreach and policy planning.

### 4.4 Role of geospatial DSS in climate-resilient agriculture

The Spatial Suitability and Forage Management Decision Support System (SSFM-DSS) created through this study exemplifies how geospatial decision tools can enhance climate-resilient agriculture. By providing detailed information about SL suitability and biomass estimates, the SSFM-DSS can help farmers and land managers make informed decisions about forage management, even in the face of climate variability. The natural tolerance of SL to drought and soil acidity makes it a valuable forage option for marginal lands—areas increasingly expanding due to soil degradation and climate variability. Such tools are not isolated. In Ethiopia, spatial DSS platforms are used to model erosion-prone zones (Hajisheko et al., 2025), while in Greece, biodiversity-focused systems guide sustainable cropping strategies (Avanidou et al., 2023). These global parallels underscore how location-specific decision tools, backed by robust geospatial analysis, can guide sustainable land management under changing environmental conditions. Compared to existing DSS platforms such as AgMod, CropSyst, or DSSAT, the SSFM-DSS is specifically tailored to a niche but critical forage legume, Sericea lespedeza and integrates both machine learning and participatory expert scoring to generate spatial recommendations. Unlike many crop DSS frameworks that rely heavily on predefined crop growth models, our approach combines empirical ground data with ANN-based prediction and geospatial analysis to provide site-specific recommendations. This hybrid architecture distinguishes SSFM-DSS in terms of flexibility, simplicity, and potential for field adoption.

### 4.5 Limitations and future directions

While the outcomes of this study are promising and demonstrate the model’s potential, we acknowledge several limitations that warrant consideration and offer opportunities for future research. First, the RBFN model, while accurate, functions as a “black box,” limiting the ability to interpret which input variables most strongly influence predictions. *Post hoc* analyses such as SHAP (Shapley Additive exPlanations), or sensitivity analysis, could enhance interpretability and provide clearer guidance for field management. Second, while PRISM datasets offer high-resolution climate data, they may overlook microclimatic variability, especially in topographically complex areas. Integrating data from localized sensor networks or incorporating higher-resolution satellite imagery could refine spatial predictions. Third, the current WebGIS tool enables one-way interaction; farmers can view recommendations but cannot upload site-specific feedback or observational data. Incorporating participatory features like data annotation and feedback submission could evolve the platform into a more dynamic, community-driven resource. This would align with open-source systems like GeoNode or QGIS, which support collaborative mapping and data sharing (Belcore et al., 2021; Kawung et al., 2023). In addition to these limitations, potential sources of error may arise from field-based biomass measurements, including variability in sampling consistency, plant moisture levels, and manual measurement error. Similarly, geospatial layers generated from UAV and Landsat imagery may introduce spatial mismatches or classification errors due to differences in resolution, atmospheric interference, or timing of image capture relative to field data. Furthermore, environmental heterogeneity within individual plots may not be fully represented by point-based sampling or coarse-resolution satellite data. While stratified sampling and data validation helped reduce these uncertainties, they remain relevant to the reliability and generalizability of model outputs. Finally, incorporating UAV-based imagery and IoT-enabled soil and weather sensors could provide near-real-time updates on forage health and environmental conditions. Such advancements would shift the SSFM-DSS from a static planning tool to a fully responsive, adaptive platform capable of supporting immediate decision-making and long-term sustainability planning. Future researchers may build upon this platform by extending its application to other forage crops, integrating farmer-driven data streams, or validating the SSFM-DSS across different agroecological zones and socio-economic contexts.

## 5 Summary & conclusion

This study introduces a novel Site-Specific Fodder Management (SSFM) paradigm designed to produce sericea lespedeza (SL). This method is especially advantageous for resource-constrained farmers in developed and developing countries, as it utilizes scalable technologies to improve fodder yield and sustainability. The study highlights agriculture’s increasing capacity to adapt to environmental stressors and the rapid advancement of technology through the integration of SSFM into SL production. This research significantly contributes to sustainable fodder management by empowering farmers with the use of modern GIS technology. This encompasses Geographic Information Systems (GIS), remote sensing, and Global Navigation Satellite Systems (GNSS), collectively offering a comprehensive framework for analyzing SL’s spatial growth dynamics, nutritional value, and biomass prediction capabilities. These tools facilitate accurate land-use planning and resource distribution decision-making, particularly in areas experiencing ecological deterioration or land shortages, giving farmers a sense of control over their operations. The growing significance of small ruminant livestock production is especially evident in regions such as the southeastern United States, where restricted land availability and changing climatic conditions are prompting a shift towards sheep and goat farming. Under this context, SL is gaining acknowledgment for its capacity to flourish under adverse environmental conditions, such as drought and low soil fertility, as well as for its superior nutritional content and established anti-parasitic capabilities. This reassessment establishes SL as a viable alternative fodder crop with considerable agronomic and ecological significance.

This study’s significant accomplishment is reclassifying SL from an invasive species to a useful and multifunctional agricultural resource. This change in perception is substantiated by a comprehensive investigation of its chemical composition, adaptability, and performance in stressful settings. The study delineates a systematic SSFM-based Decision Support System (DSS) to improve animal productivity in drought-affected and marginally productive regions, offering a hopeful outlook for farmers in challenging conditions. The study’s hallmark is using automated geospatial modeling tools and developing an interactive WebGIS Dashboard. This digital platform democratizes access to precision agriculture technologies, making farmers feel included in a progressive movement. It enables the distribution of spatial information and allows farmers to make site-specific management decisions by assessing essential factors such as soil stability, moisture availability, nutrient levels, and local climatic patterns. This innovation improves operational efficiency and democratizes access to precision agriculture technologies.

Furthermore, the research underscores SL’s diverse function in contemporary agriculture as a robust, nutrient-dense forage crop and a fundamental component of sustainable livestock systems. Its intrinsic anti-parasitic properties and plasticity render it a pivotal choice for incorporating ecological resilience into animal feeding regimens, especially in low-input systems. The effective execution of the SSFM-DSS model illustrates its scalability and applicability across various agroecological zones. This study advocates amalgamating SSFM principles with advanced technical tools to establish a more sustainable, productive, and climate-resilient agricultural environment. The approach demonstrates potential for implementation in many socio-economic and geographic contexts, providing substantial advantages to smallholders and commercial farmers globally.

## Data Availability

The raw data supporting the conclusions of this article will be made available by the authors, without undue reservation.
